# Supplementary material for: Triazole Stress Induces Accelerated Degradation of Aspergillus fumigatus Hmg1
Source: bioRxiv. 2026 Oct 3:2026.10.02.756251. Preprint. [Version 1] doi: 10.64898/2026.10.02.756251 (PMC13682438; doi:10.64898/2026.10.02.756251)

Supplemental Figure 1

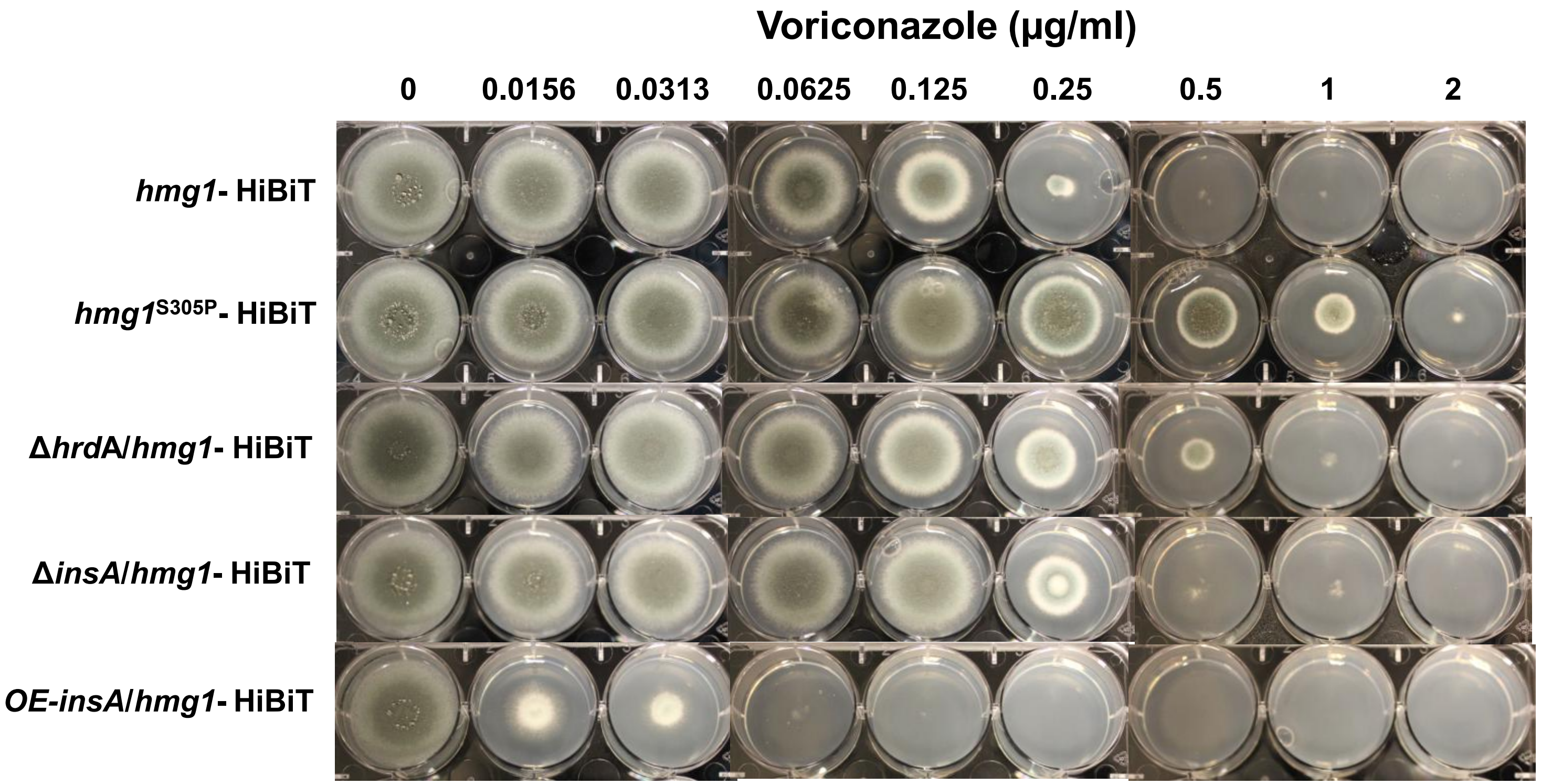

Supplemental Figure 2

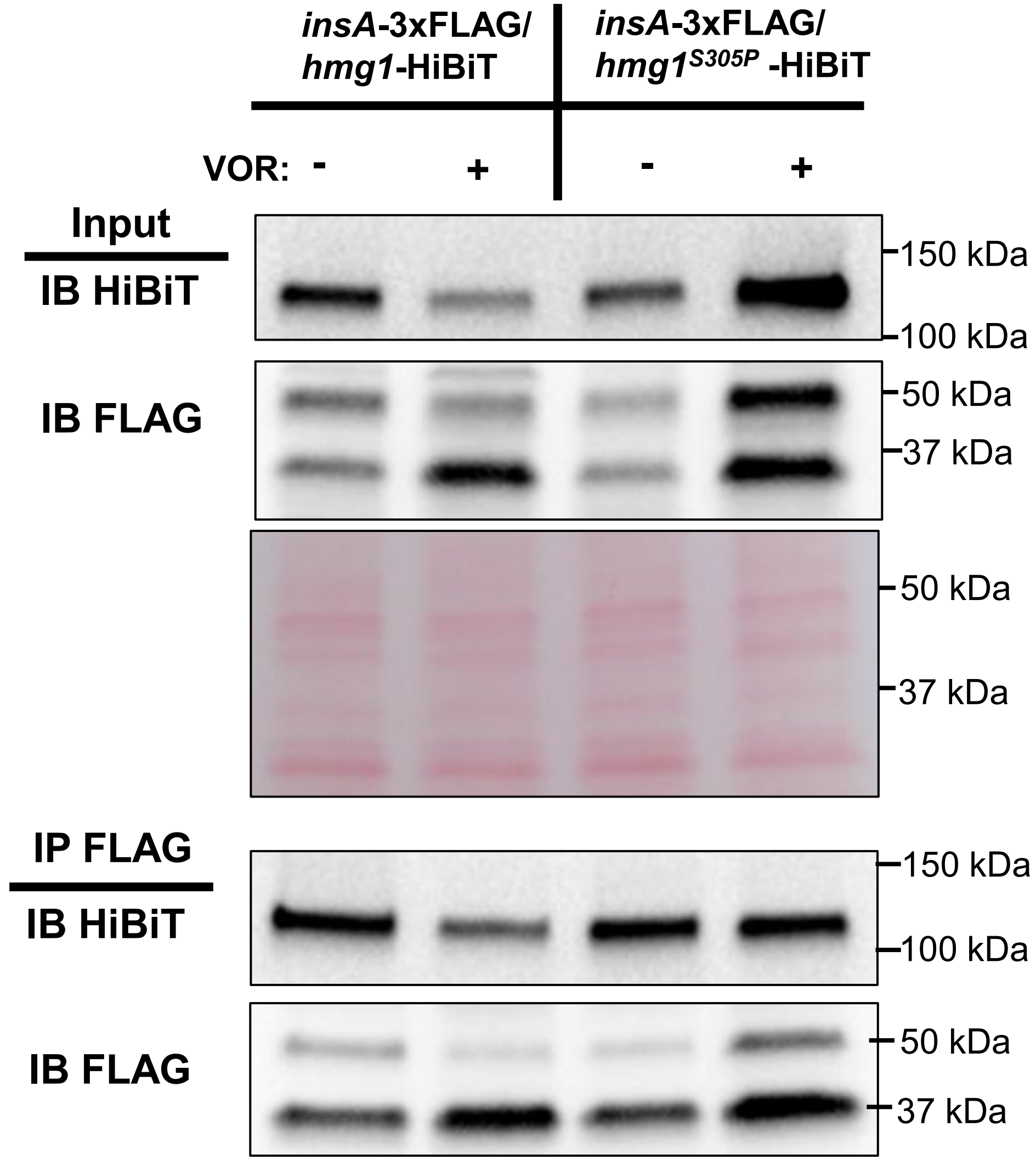

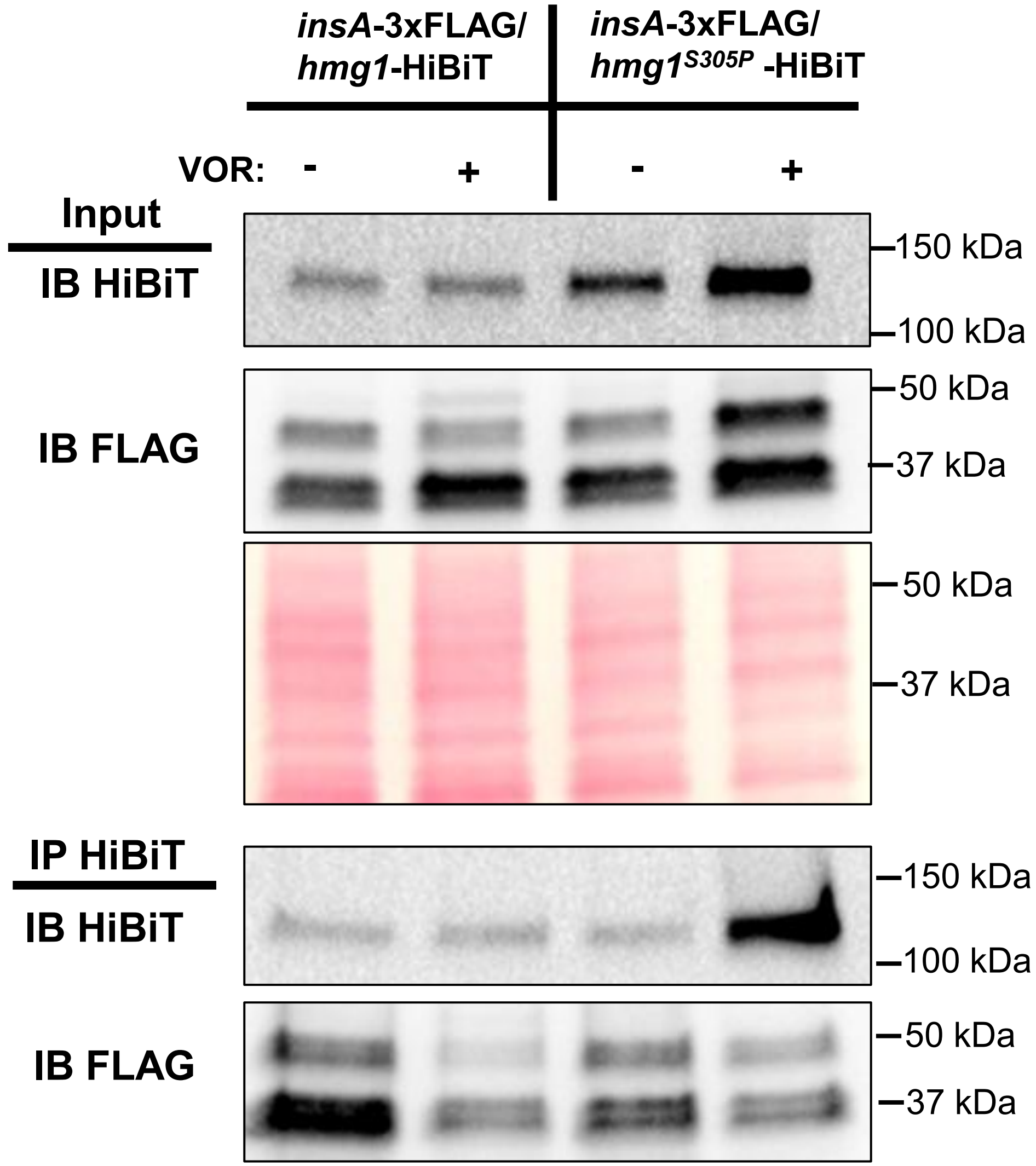

Supplemental Figure 3

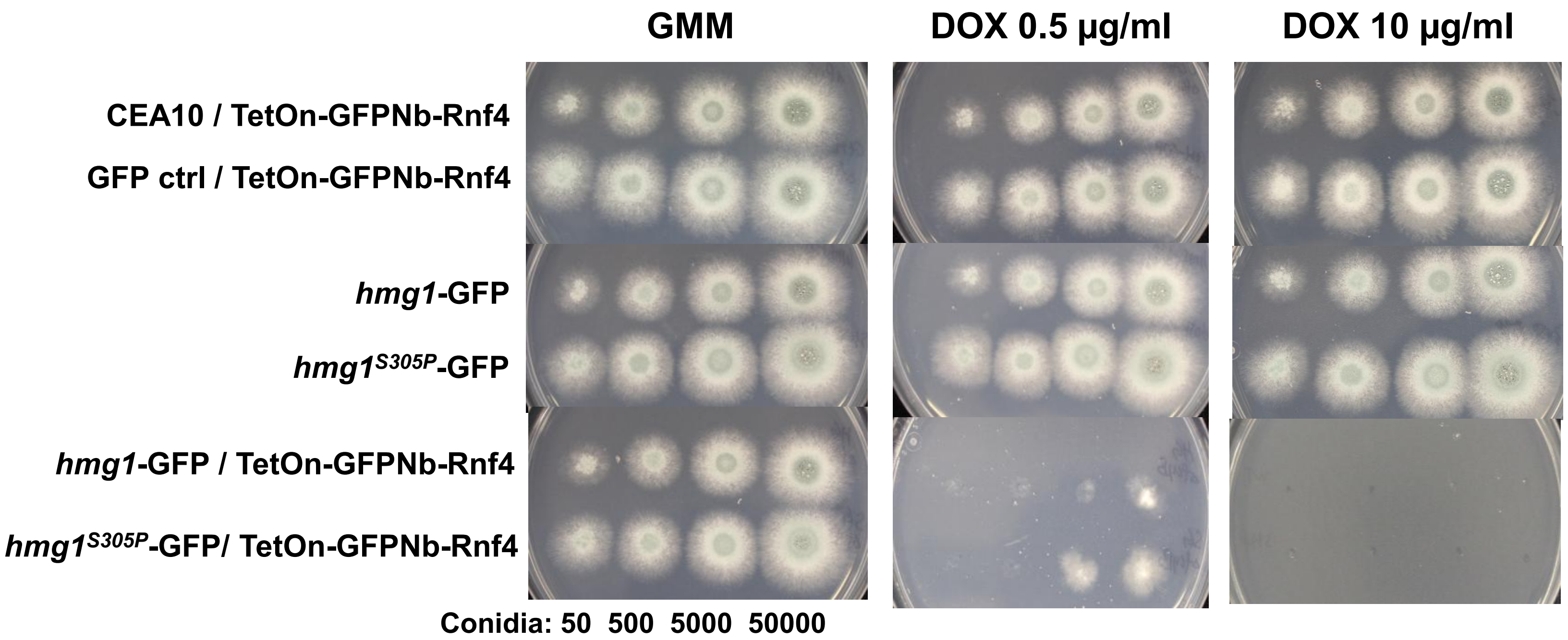

Supplement: Supplementary file 1 [file NIHPP2026.10.02.756251V1-supplement-1.pdf]
